# Super-enhancers and the super-enhancer reader BRD4: tumorigenic factors and therapeutic targets

**DOI:** 10.1038/s41420-023-01775-6

**Published:** 2023-12-22

**Authors:** Haihong Qian, Min Zhu, Xinyu Tan, Yixing Zhang, Xiangning Liu, Li Yang

**Affiliations:** 1 Yangpu Hospital, School of Medicine, Tongji University, Shanghai, 200090 China; 2 Department of Dentistry, Kunming Medical University, Kunming, 650032 China

**Keywords:** Targeted therapies, Oncogenes

## Abstract

Transcriptional super-enhancers and the BET bromodomain protein BRD4 are emerging as critical drivers of tumorigenesis and therapeutic targets. Characterized by substantial accumulation of histone H3 lysine 27 acetylation (H3K27ac) signals at the loci of cell identity genes and critical oncogenes, super-enhancers are recognized, bound and activated by BRD4, resulting in considerable oncogene over-expression, malignant transformation, cancer cell proliferation, survival, tumor initiation and progression. Small molecule compound BRD4 BD1 and BD2 bromodomain inhibitors block BRD4 binding to super-enhancers, suppress oncogene transcription and expression, reduce cancer cell proliferation and survival, and repress tumor progression in a variety of cancer types. Like other targeted therapy agents, BRD4 inhibitors show moderate anticancer effects on their own, and exert synergistic anticancer effects in vitro and in preclinical models, when combined with other anticancer agents including CDK7 inhibitors, CBP/p300 inhibitors and histone deacetylase inhibitors. More recently, BRD4 BD2 bromodomain selective inhibitors, proteolysis-targeting chimera (PROTAC) BRD4 protein degraders, and dual BRD4 and CBP/p300 bromodomain co-inhibitors have been developed and shown better anticancer efficacy and/or safety profile. Importantly, more than a dozen BRD4 inhibitors have entered clinical trials in patients with cancer of various organ origins. In summary, super-enhancers and their reader BRD4 are critical tumorigenic drivers, and BRD4 BD1 and BD2 bromodomain inhibitors, BRD4 BD2 bromodomain selective inhibitors, PROTAC BRD4 protein degraders, and dual BRD4 and CBP/p300 bromodomain co-inhibitors are promising novel anticancer agents for clinical translation.

## Introducton

Transcriptional enhancers are short regulatory DNA elements which bind RNA polymerase II (RNA Pol II), transcription factors and co-regulators, and are characterized by acetylated histone H3 lysine 27 (H3K27ac) and monomethylated H3K4 (H3K4me) signals in chromatin immunoprecipitation sequencing assays [1]. As enhancers can form loops with promoters over a long distance, enhancers augment the transcription of neighboring genes, irrespective of the sense or antisense direction of their target genes [2, 3].

Super-enhancers are large clusters of enhancers that are in close genomic proximity, are densely bound by the BET bromodomain protein BRD4 and master transcription factors, and are characterized by massive H3K27ac and H3K4me signals in ChIP sequencing [4–6].

## Enhancers activate gene transcription and induce tumorigenesis

**Fig. 1:**
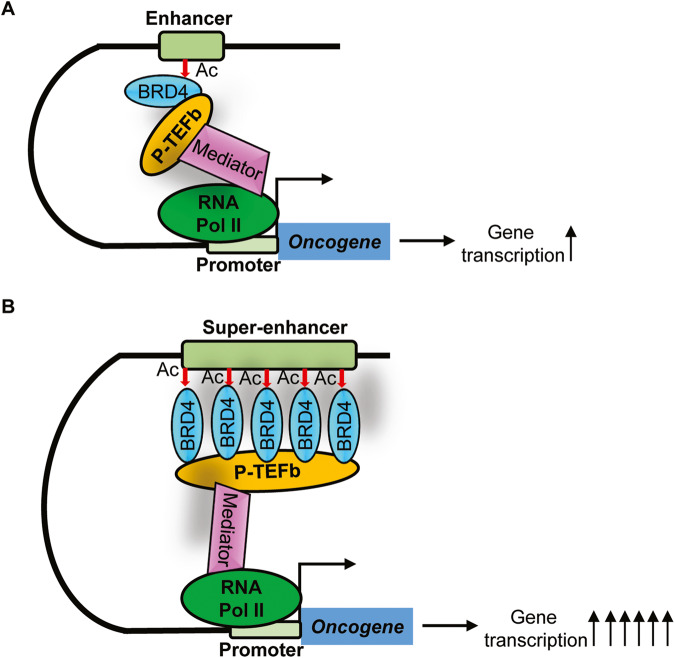
Transcriptional enhancers and super-enhancers activate gene transcription. **A**, **B** The BET bromodomain protein BRD4 recognizes acetylated (Ac) histone H3 lysine 27, binds to and activates enhancers (**A**) and super-enhancers (**B**). BRD4 recruits the positive transcription elongation factor b (P-TEFb) and Mediator, leading to RNA Polymerase II (RNA Pol II) activation and binding to enhancer- and super-enhancer-associated gene promoter, transcriptional activation and target gene over-expression. As super-enhancers are bound by much larger clusters of BRD4 proteins, super-enhancer-associated oncogenes are transcribed at substantially higher levels than enhancer-associated genes.

Transcriptional enhancers recruit BRD4, transcription factors and cofactors to activate RNA Pol II and gene transcription from gene promoters [7] (Fig. 1A). Transcriptional enhancers have been confirmed to play an important role in the activation and over-expression of oncogenes, such as *MYC* which is juxtaposed to the immunoglobulin heavy-chain gene enhancer in Burkitt’s lymphoma [8].

The Hippo pathway transcription coactivators YAP/TAZ form a protein complex with TEAD and AP-1 at distal transcriptional enhancers rather than promoters, located >100,000 base pairs away from transcription start sties. Through chromatin looping, the YAP/TAZ/TEAD/AP-1 transcription cofactor and transcription factor complex activate the transcription of enhancer-associated genes including those controlling S-phase entry and mitosis of the cell cycle, resulting in cell proliferation and skin tumorigenesis [9]. The oncogenic transcription factor FOXA1 is hyperactive in metastatic endocrine-resistant breast cancer cells due to gene amplification or overexpression. FOXA1 induces enhancer reprogramming and transcriptional activation of pro-metastatic oncogenes in endocrine-resistant breast cancer cells [10].

The transcriptional activator NRF2 is frequently activated in non-small cell lung cancer, and NRF2 overexpression results in the accumulation of CCAAT Enhancer Binding Protein Beta (CEBPB) [11]. NRF2 and CEBPB co-operatively induce the establishment of transcriptional enhancers at the loci of oncogenes such as the *NOTCH3* gene [11]. Importantly, in mouse models of non-small cell lung cancer, disruption of the *NOTCH3* enhancer significantly suppresses tumor progression and augments the anticancer effects of cisplatin, demonstrating the important role of the *NOTCH3* enhancer in tumorigenesis and drug resistance [11].

Recent transcriptome profiling has shown that squamous cell lineage markers are present in ~25% of pancreatic ductal adenocarcinoma tumors, and the squamous cell subtype is associated with poorer patient prognosis [12]. Aberrant enhancers have recently been found to be established in the squamous cell subtype of pancreatic ductal adenocarcinoma tumors. Enhancers at the loci of oncogenes, such as *MYC* and *HRAS*, play a critical role in pancreatic ductal adenocarcinoma cell transition into squamous cells, cell migration and invasion in vitro, and accelerated tumor growth and metastases in vivo [13].

### Enhancers can also activate tumor suppressor gene transcription and suppress tumorigenesis

Enhancers can also activate tumor suppressor gene transcription and thereby suppress tumorigenesis. The N-terminal SNAG domain of the transcriptional repressor GFI1 binds to the CoREST transcriptional complex proteins LSD1 and RCOR1 at the enhancers of transcription factor genes, such as *SPI1* (PU.1), *CEBPA* and *IRF8* which are important for acute myeloid leukemia cell differentiation [14]. GFI1 inactivation or LSD1 inhibition with small molecule compound inhibitors disrupts the interaction between GFI1, LSD1 and RCOR1, leading to considerable increase in H3K27ac at enhancer regions of the transcription factor genes, transcriptional activation, acute myeloid leukemia cell differentiation, growth inhibition and clonogenicity reduction [14].

SWI/SNF (mSWI/SNF or BAF) chromatin remodeling complex inactivation contributes to >20% of human cancers. Forced over-expression of the core BAF complex subunit SMARCB1 in sarcoma cells results in the activation of distal typical enhancers and super-enhancers at the loci of genes such as *CDKN1A* [15]. The activated typical enhancers and super-enhancers play critical roles in sarcoma cell growth arrest, demonstrating a tumor suppression effect [15]. Therefore, enhancers can induce or suppress tumorigenesis, probably depending on cancer subtypes and cellular contexts.

### Super-enhancers activate oncogene transcription and induce tumorigenesis

Super-enhancers consist of enhancer clusters, span large genetic regions, and are generally an order of magnitude larger than typical enhancers [4, 5]. Super-enhancers are bound by a large number of BRD4 which recruits the Mediator, a protein complex connecting the transcription factors at the super-enhancers and RNA pol II at the gene promoters (Fig. 1B) [4, 5].

Super-enhancers are emerging as critical regulators of oncogene transcription and tumorigenesis. In glioblastoma cells, super-enhancers have been found to be associated with a number of oncogenic genes, such as *RUNX1*, *BCL3* and *FOSL2* [6]. In glioblastoma stem cells isolated from PDX mouse models originally derived from human tumor samples, a subset of super-enhancers at the loci of critical genes, such as *CDK6*, *SOX2*, *EGFR* and *BRD4*, are shared by the majority of human glioblastoma stem cells [16]. Proximity of the super-enhancers to their associated genes correlates with gene over-expression in glioblastoma stem cells and human tumor samples, and the core glioblastoma stem cell super-enhancer-associated genes are essential for glioblastoma cell proliferation and tumorigenesis [16] (Table 1). In addition, patients with glioblastoma that is enriched of the core glioblastoma stem cell super-enhancer signature show more advanced tumor stage and poorer prognosis [16].

**Table 1:** Super-enhancers and BRD4 induce oncogene transcriptional activation and over-expression, cancer cell proliferation, survival, tumor initiation and progression.

Cancer type	Regulation of gene expression	Regulation of tumorigenesis	References
Glioblastoma	*RUNX1*, *BHLHE40, BCL3*, *FOSL2, EGFR* and *SOX2* gene over-expression	Glioblastoma cell proliferation in vitro and tumor progression in mice	[6, 16]
Liver cancer	*SPHK1*, *MYC*, *MYCN*, *SHH* and *YAP1* oncogene over-expression	Liver cancer cell proliferation in vitro and tumor progression in mice	[17]
Pancreatic cancer	*MYC* and *RUNX3* oncogene over-expression	Pancreatic cancer cell de-differentiation, proliferation in vitro and tumor progression in a mouse model	[18]
Small cell lung cancer	Lineage-specific transcription factor and *MYC, SOX2* and *NFIB* gene over-expression	Small cell lung cancer cell proliferation in vitro and tumor progression in mouse models	[19]
Neuroblastoma	*MYC*, *MYCN* and *JMJD6* oncogene over-expression	Neuroblastoma cell proliferation in vitro and tumor progression in mice	[20]
Clear cell renal cell carcinoma	*CXCL1*, *CXCL5* and *CXCL8* CXC chemokine gene over-expression	Renal cell cancer progression and metastasis in mice	[22]
Colon cancer	*ASCL2*, *PDZK1IP1* and *MYC* over-expression	Colon cancer cell proliferation in vitro and tumor progression in mice	[23]
Medulloblastoma	*ALK*, *SMO*, *NTRK3*, *LMO1 LMO2*, *MYC*, *ETV4* and *PAX5* over-expression	Medulloblastoma cell proliferation in vitro and tumor progression in mice	[26, 48]
Leukemia	*MYC* over-expression	leukemia stem cell self-renewal in vitro and leukemogenesis	[28, 29]
Diffuse large B cell lymphoma	*MYC*, *E2F1*, *BCL6* and *PAX5* oncogene over-expression	Lymphoma cell proliferation in vitro and lymphoma progression in mice	[34]
Estrogen receptor alpha positive breast cancer	Over-expression of estrogen receptor alpha target genes, such as *RET*	Estrogen receptor alpha positive breast cancer cell proliferation in vitro and tumor progression in mice	[35]
Neck & nasopharyngeal squamous cell carcinoma	Over-expression of oncogenes such as *ETV6*, and cancer stemness genes such as *MET*, *TP63* and *FOSL1*	Cancer cell proliferation and cancer stem cell self-renewal in vitro, and invasive tumor growth and metastasis in mice	[36, 37]
Diffuse intrinsic pontine glioma	Over-expression of *oncogenes such as EGFR* & undifferentiation genes such as *SOX2* and *NES*	Diffuse intrinsic pontine glioma cell proliferation in vitro and tumor progression in mouse models	[21]
Rhabdomyosarcoma	*SOX8*, *MYOD1*, *MYOG* and *MYCN* over-expression	Rhabdomyosarcoma cell proliferation in vitro and tumor growth in mice	[38, 39]
Melanoma	*PGC*-*1α* gene over-expression	Melanoma cell proliferation in vitro and tumor growth in a mouse model	[40]
Multiple myeloma	*HJURP, MYC, BCL-xL* and *IRF4* gene over-expression	Multiple myeloma cell proliferation and survival	[6, 41]

Super-enhancers are extensively reprogrammed during liver cancer tumorigenesis [17]. Liver cancer cells acquire super-enhancers at the loci of critical oncogenic genes, such as *SPHK1*, *MYC*, *MYCN*, *SHH* and *YAP1*, to drive their substantial over-expression. The super-enhancer “writer” p300, super-enhancer “reader” BRD4, and super-enhancer activity regulators CDK7 and MED1 are often over-expressed in human liver cancer tissues, and their over-expression predicts poor patient prognosis [17]. Importantly, inhibition of p300, BRD4, CDK7 or MED1 reduces the expression of super-enhancer-associated oncogenes and exerts anticancer effects against liver cancer [17].

The histone demethylase *KDM6A* gene is often mutated in a variety of human malignancies. Loss of function of *KDM6A* causes squamous-like metastatic pancreatic cancer through aberrant activation of super-enhancers at the loci of *MYC* and *RUNX3* oncogenes and consequent *MYC* and *RUNX3* over-expression [18]. Treatment with BRD4 inhibitors results in *KDM6A* mutant pancreatic cancer cell differentiation and tumor growth inhibition in a mouse model [18] (Table 1).

The super-enhancer landscape of small cell lung cancer cells recapitulates embryonic, neural and tumorigenic signatures, as many super-enhancers are associated with lineage-specific transcription factor genes and oncogenes such as *MYC, SOX2* and *NFIB* [19] (Table 1). In a high-throughput compound screening, small cell lung cancer cells have been found to be very sensitive to the CDK7 inhibitor THZ1 which selectively suppresses the expression of super-enhancer associated genes [19].

In chromosome 17q-gained neuroblastoma, the *JMJD6* gene is over-expressed due to both gene gain and transcriptional super-enhancers, and suppression of super-enhancer activity reduces *JMJD6* gene expression, neuroblastoma cell proliferation in vitro and tumor growth in a mouse model [20] (Table 1). Similarly, in diffuse intrinsic pontine glioma, the expression of critical oncogenic genes such as *SOX2* and *NOTCH1* is regulated by super-enhancers, and treatment with super-enhancer inhibitors reduces diffuse intrinsic pontine glioma cell proliferation in vitro and tumor progression in mouse models [21] (Table 1).

In clear cell renal cell carcinoma, super-enhancers are formed at the loci of CXC chemokine genes, such as *CXCL1*, *CXCL5* and *CXCL8*, and induce CXC chemokine gene over-expression and renal cell carcinoma progression and metastasis [22]. Consistent with these findings, suppression of super-enhancer activity reduces CXC chemokine gene expression and renal cell cancer metastasis [22] (Table 1).

Compared with normal counterparts, colon cancer cells gain oncogenic super-enhancers, including super-enhancers associated with *ASCL2*, a transcription factor for intestinal stem cell fate, and the Wnt target gene *MYC* [23]. In addition, β-catenin and CTCF up-regulate *MYC* by connecting nucleoporins to oncogenic super-enhancers, leading to MYC mRNA export to the cytoplasm, stabilization and over-expression [24, 25]. Interestingly, inflammation in the tumor microenvironment results in the formation of super-enhancers at the *PDZK1IP1* gene locus, resulting in colon cancer cell proliferation in vitro and tumor progression in a mouse model [23] (Table 1).

Medulloblastoma are divided into 4 distinct groups, WNT, SHH, Group 3, and Group 4 groups, and the 4 different groups show distinct super-enhancer profiles. Association of critical oncogenes with super-enhancers has been found at the *ALK* gene locus in WNT group, at *SMO* and *NTRK3* gene loci in SHH group, at the *LMO1*, *LMO2* and *MYC* gene loci in Group 3, and at the *ETV4* and *PAX5* gene loci in Group 4 [26] (Table 1).

In leukemic stem cells, the *MYC* gene locus is characterized by super-enhancers which recruit critical transcriptional factors including MYB, RUNX1 and GFI1b to drive *MYC* over-expression and leukemogenesis [27]. In chronic myelogenous leukemia stem cells, suppression of super-enhancer-driven gene transcription by a CDK7 inhibitor eradicates leukemia stem cells in a mouse model without effects in normal hematopoietic stem cells [28]. In human primary T cell acute lymphoblastic leukemia samples, a topologically associating domain ‘fusion’ event due to CTCF-mediated insulation absence results in the interaction between distal super-enhancers and the *MYC* gene promoter, leading to *MYC* over-expression and leukemogenesis [29] (Table 1).

Super-enhancers have also been shown to be important in epithelial-to-mesenchymal transition (EMT) and metastasis. *ETS2*, *JUNB, EGFR* and *HNF4A* genes are associated with super-enhancers in non-small cell lung cancer cells. Suppression of super-enhancer activity reduces the expression of these super-enhancer-associated genes, decreases non-small cell lung cancer cell migration and invasion, and abrogates TGF-β-induced EMT, demonstrating the role of super-enhancers in regulating EMT and tumor metastasis [30].

### Super-enhancers can function as tumor suppressors

While generally proven to promote tumor initiation and progression, super-enhancers can also function as tumor suppressors. The histone methyltransferase *KMT2D* is often inactivated in human lung cancer tissues. Loss of *KMT2D* reduces the activity of super-enhancers at critical genes, such as the circadian rhythm repressor *Per2*, resulting in *Per2* gene down-regulation, glycolysis and lung cancer tumorigenesis [31]. In breast cancer, loss of the tumor suppressor gene *RCAN1.4* augments tumor metastasis. Unexpectedly, *RCAN1.4* gene expression is driven by super-enhancers in breast cancer cells, and suppression of super-enhancer activity with BRD4 knockdown or BRD4 inhibitor treatment reduces *RCAN1.4* tumor suppressor gene expression [32].

The super-enhancer “reader” BRD4 forms a protein complex with the repressive LSD1/NuRD transcription regulators at super-enhancers to suppress the expression of drug resistance genes in breast cancer cells [33]. Repression of super-enhancer activity with BRD4 inhibitors does not have an immediate effect on the expression of the drug resistance genes, however, long-time treatment with BRD4 inhibitors causes resistance to both BRD4 inhibitors and a broad spectrum of anticancer agents, demonstrating the role of super-enhancers and BRD4 in super-enhancer-mediated transcriptional repression of genes involved in tumorigenesis and chemoresistance [33]. Therefore, long-term treatment with BRD4 inhibitors might promote multidrug resistance and tumor progression, and close monitoring and prompt intervention are required in clinical trials.

## The super-enhancer “reader” Brd4 promotes super-enhancer-associated oncogene transcription and tumorigenesis and Brd4 inhibitors exert anticancer effects

**Fig. 2:**
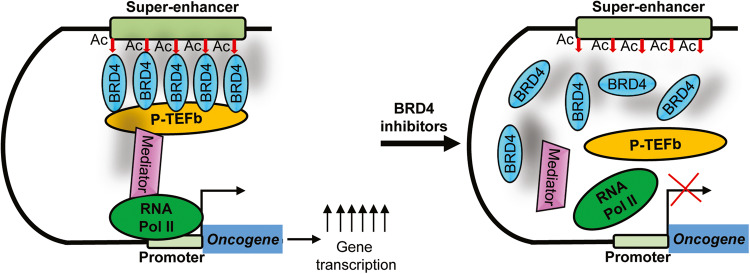
BRD4 inhibitors suppress oncogene transcription and expression. The BET bromodomain protein BRD4 recognizes acetylated (Ac) histone H3 lysine 27, binds to and activates super-enhancers, leading to RNA Polymerase II (RNA Pol II) binding to super-enhancer-associated oncogene promoter, gene transcriptional activation and over-expression. Treatment with BRD4 inhibitors displaces BRD4 at super-enhancers, leading to RNA Pol II disassociation from gene promoters and transcriptional suppression.

The BET bromodomain protein BRD4 recognizes, binds to and activates super-enhancers and substantially up-regulate the expression of super-enhancer-associated oncogenes (Fig. 1B), and BRD4 inhibitors blocks BRD4 binding and reduce oncogene expression (Fig. 2). In diffuse large B cell lymphoma, approximately one-third of BRD4 protein localizes to super-enhancers which occupy ~1.6% of genes [34]. Treatment with four different BRD4 inhibitors reduces the expression of super-enhancer-associated oncogenes, such as *MYC*, *E2F1*, *BCL6* and *PAX5*, and reduces diffuse large B cell lymphoma cell proliferation. Treatment of mice xenografted with diffuse large B cell lymphoma with the BRD4 inhibitor JQ1 suppresses lymphoma progression [34] (Table 1).

In estrogen receptor alpha (ERα)-positive breast cancer cells, BRD4 is a master activator of ERα-occupied super-enhancers and the transcription of ERα target genes, such as *RET* which in turn activates ERα phosphorylation and ERα target gene expression. BRD4 therefore induces breast cancer cell proliferation and tumor progression [35] (Table 1).

In human neck squamous cell carcinoma, BRD4 recruits Mediators and NF-κB at super-enhancers associated with cancer stemness genes such as *MET*, *TP63* and *FOSL1*. Treatment with BRD4 inhibitors reduces stemness gene expression; suppresses cancer stem cell self-renewal, invasive growth and metastasis; and eliminates tumor cells and cancer stem cells in a mouse model of neck squamous cell carcinoma [36]. In nasopharyngeal carcinoma cells, super-enhancers are enriched of BRD4, NF-κB, IRF1 and IRF2 transcription factors at the loci of critical oncogenes such as *ETV6*, high expression of which in human nasopharyngeal carcinoma tissues is correlated with poor patient prognosis [37]. Treatment with the BRD4 inhibitor JQ1 significantly suppresses super-enhancer-associated *ETV6* gene expression and induces nasopharyngeal carcinoma cell growth inhibition [37] (Table 1).

In diffuse intrinsic pontine glioma, super-enhancers are found at the loci of a number of genes indicating undifferentiation status such as *SOX2* and *NES* as well as oncogenes such *EGFR* [21]. These super-enhancers are characterized by BRD4 binding, and BRD4 knockdown or inhibition reduces diffuse intrinsic pontine glioma cell proliferation in vitro and tumor progression in mouse models [21] (Table 1).

In rhabdomyosarcoma, super-enhancers are bound by core regulatory transcription factors and are characterized by the highest levels of histone acetylation [38]. Counterintuitively, the super-enhancers are also bound by the most histone deacetylases (HDACs), and HDAC inhibitors augment BRD4, but decreases RNA Pol II and core regulatory transcription factor, binding to the super-enhancers. The data demonstrate super-enhancer-specific requirement to balance histone acetylation and deacetylation for maintaining super-enhancer architecture and gene transcription [38]. In alveolar rhabdomyosarcoma, the chimeric transcription factor PAX3-FOXO1 interacts with the master transcription factors MYCN, MYOG and BRD4 at target gene super-enhancers, resulting in over-expression of *SOX8*, *MYOD1*, *MYOG* and *MYCN*, alveolar rhabdomyosarcoma tumorigenesis and dependence on BRD4 [39]. Inhibition of BRD4 with the BRD4 inhibitor JQ1 or OTX015 abolishes PAX3-FOXO1 function, suppresses alveolar rhabdomyosarcoma cell proliferation in vitro and induces tumor growth inhibition in mouse models [39] (Table 1).

Melanoma with PGC-1α over-expression is characterized by substantial BRD4 protein binding at the *PGC*-*1α* gene super-enhancer [40]. Treatment with the BRD4 inhibitor JQ1 or BAY 1238097 blocks BRD4 binding to the super-enhancer and PGC-1α expression, suppresses melanoma cell proliferation in vitro, and inhibits tumor growth in a mouse model [40] (Table 1).

In multiple myeloma, BRD4 and Mediator are enriched at super-enhancers associated with oncogenes including *MYC, BCL-xL* and *IRF4*. Treatment of multiple myeloma cells with the BRD4 inhibitor JQ1 results in BRD4 disassociation from super-enhancers, and reduction in *MYC, BCL-xL* and *IRF4* gene expression and multiple myeloma cell proliferation [6]. In t(4;14)-positive multiple myeloma, BRD4 interacts with the histone lysine methyltransferase NSD2 at the *HJURP* gene super-enhancers, leading to *HJURP* gene over-expression, multiple myeloma cell proliferation and survival [41] (Table 1). Taken together, BRD4 promotes super-enhancer-associated oncogene transcription and tumorigenesis, and BRD4 inhibitors exert anticancer effects.

## Small molecule compound Brd4 inhibitors and degraders exert promising anticancer effects in pre-clinical models

### Small molecule compound BRD4 BD1 and BD2 bromodomain inhibitors in cancer therapy

In the past decade, a number of small molecule compound BRD4 inhibitors have been developed through chemical synthesis, structure-based in silico screen, and wet lab screen of small molecule compound libraries. The majority of the inhibitors, such as JQ1, OTX015, I-BET762, MK-8628, NHWD870, ABBV-744, PLX2853 and INCB054329, target both the BD1 and BD2 bromodomains of BRD4, reduce oncogene expression, and exert anticancer effects in pre-clinical models.

Pancreatic ductal adenocarcinoma, head and neck squamous cell carcinoma and leukemia are characterized by oncogene over-expression due to super-enhancers. Combination therapy with the BRD4 inhibitor JQ1 and the CDK7 inhibitor THZ1 synergistically reduces super-enhancer-associated oncogene expression and exerts synergistical anticancer effects against pancreatic ductal adenocarcinoma and head and neck squamous cell carcinoma in vitro and in mouse models [42, 43]. Interestingly, combination therapy with BRD4 inhibitors and CDK7 inhibitors overcomes resistance to BRD4 inhibitor therapy in leukemia cells and mouse models of leukemia [44]; and nanoparticle-mediated delivery of JQ1 and THZ1, compared with free drug formulation, considerably reduces cytotoxicity to liver cells but synergistically suppresses tumor progression in a mouse model of drug-resistant pancreatic ductal adenocarcinoma [42].

CDK4/CDK6 inhibitors have also been shown to exert synergistic anticancer effects with BRD4 inhibitors in castration-resistant prostate cancer and NUT midline carcinoma [45, 46]. Castration-resistant prostate cancer cells with high levels of the deubiquitinase DUB3 and NUT midline carcinoma cells with high levels of KLF4 are resistant to BRD4 inhibitors, because DUB3 binds to BRD4 and augments its deubiquitination and stabilization and KLF4 up-regulates *E2F* and *MYC* gene expression [45, 46]. As DUB3 is activated after phosphorylation by CDK4 and CDK6 and *E2F* and *MYC* expression are activated after Rb phosphorylation by CDK4 and CDK6, treatment with the CDK4/CDK6 inhibitor Palbociclib sensitizes prostate cancer and NUT midline carcinoma cells to the BRD4 inhibitor JQ1, and exerts synergistic anticancer effects with JQ1 in vitro and in mouse models of castration-resistant prostate cancer and NUT midline carcinoma [45, 46].

In a high-throughput drug screen, BRD4 inhibitors have been found to be one of the two classes of compounds exerting the best synergistic anticancer effects with the CDK4/CDK6 inhibitor Ribociclib in medulloblastoma cells [47]. A reverse combination drug screen identifies CDK4/CDK6 inhibitors as the compounds exerting the best synergy with the BRD4 inhibitor JQ1 against medulloblastoma cells [47]. Treatment with the orally bioavailable BRD4 inhibitor MK-8628 suppresses medulloblastoma cell proliferation and induces apoptosis by reducing *MYC* expression, and MK-8628 suppresses medulloblastoma tumor progression in preclinical models [48]. Co-treatment with MK-8628 and the PLK1 inhibitor Volasertib, which targets MYC protein for degradation, shows synergistic anti-medulloblastoma effects in vitro and in preclinical models [48].

Another well-studied anticancer agent for BRD4 inhibitor combination therapy is HDAC inhibitors, particularly the pan-HDAC inhibitor Panobinostat. Combination therapy with the BRD4 inhibitor JQ1 or OTX015 and Panobinostat synergistically reduces the expression of oncogenes, such as *MYC*, *MYCN* and *LIN28B*; suppresses proliferation and induces apoptosis in *MYCN* gene-amplified neuroblastoma, medulloblastoma and diffuse intrinsic pontine glioma cells; and significantly suppresses neuroblastoma and medulloblastoma tumor progression in mouse models [21, 49, 50]. In neuroblastoma due to *TERT* gene rearrangement with super-enhancers, BRD4 is required for *TERT* gene transcription and neuroblastoma cell proliferation [51]. In an unbiased screen of approved oncology drugs, the BRD4 inhibitors I-BET762 and OTX015 exert the best synergistic anticancer effects with the proteasome inhibitor Carfilzomib; and OTX015 and carfilzomib synergistically reduce *TERT* expression, induces *TERT* gene-rearranged neuroblastoma cell apoptosis, blocks tumor progression and improves survival in multiple mouse models of *TERT* gene-rearranged neuroblastoma [51].

Unbiased high-throughput drug combination screens reveal that PI3K-AKT-mTOR pathway inhibitors exert synergistic anticancer effects with BRD4 inhibitors against small cell lung cancer cells, and mTOR inhibitors exhibit the best synergy [52]. Mechanistically, while BRD4 inhibitors up-regulate RSK3 to activate the mTOR pathway, mTOR inhibitors block this cell survival signaling and enhance BRD4 inhibitor-mediated cancer cell apoptosis [52]. In multiple patient-derived xenograft models of small cell lung cancer, combination therapy with the mTOR inhibitor Everolimus and the BRD4 inhibitor NHWD870 synergistically induce cancer cell apoptosis and blocks tumor progression without significantly increasing toxicity to normal tissues in mice [52]. In Ewing sarcoma cell lines and patient-derived xenograft (PDX) lines, AKT pathway activation protects Ewing sarcoma cells against BRD4 inhibitors, and IGF1R inhibitors and mTOR inhibitors suppress AKT pathway activation and synergistically enhance cancer cell sensitivity to BRD4 inhibitors [53]. In PDX models of Ewing sarcoma, treatment with the BRD4 inhibitor NHWD870 and the IGF1R inhibitor BMS754807 results in substantial and durable anticancer effects, while monotherapy was much less effective [53].

Genome-wide loss-of-function clustered regularly interspaced short palindromic repeats (CRISPR) screens identify *SPOP* gene deficiency as a resistance factor to BRD4 inhibitor therapy in *KMT2A* gene-rearranged leukemia cells [54]. Kinase vulnerability CRISPR screens identify GSK3 inhibitors as effective agents to overcome *SPOP* deficiency-induced BRD4 inhibitor resistance. Combination therapy with the BRD4 inhibitor ABBV-744 and the GSK3 inhibitor CHIR-98014 considerably suppresses *KMT2A*-rearranged leukemia progression in patient-derived xenograft models in mice, confirming ABBV-744 and CHIR-98014 combination therapy as an effective therapeutic strategy [54]. Since it is now clear that targeted therapies need to be combined with other anticancer agents in the clinic to exert better anticancer effects and to reduce toxicity, the other anticancer agents should be identified by unbiased screening of anticancer drug libraries for each cancer subtype.

### Small molecule compound BRD4 BD2 selective bromodomain inhibitors in cancer therapy

While the majority of BRD4 inhibitors bind to the BD1 and BD2 bromodomains of BRD4 with similar affinities, the small molecule compound ABBV-744 selectively binds to the BD2 bromodomain [55]. By selectively suppressing the BD2 bromodomain, ABBV-744 induces acute myeloid leukemia and prostate cancer cell growth inhibition, and exhibits significant anticancer effects against acute myeloid leukemia and prostate cancer in mouse models with better toxicity profile and therapeutic index than BRD4 BD1 and BD2 bromodomain inhibitors [55, 56]. In addition, while the BRD4 inhibitors PLX2853 and INCB054329 show synergistic anticancer effects when combined with the BCL2 inhibitor Venetoclax in mouse models of diffuse large B-cell lymphoma and acute myeloid leukemia, ABBV-744 also exerts synergistic anticancer effects with Venetoclax in mouse models of acute myeloid leukemia (Table 2) [56, 57]. Interestingly, GSK620, another small molecule compound BRD4 BD2 bromodomain selective inhibitor, suppresses inflammatory disease in pre-clinical models (Table 2) [58].

**Table 2:** BRD4 BD2 bromodomain selective inhibitors, PROTAC BRD4 protein degraders, and dual BRD4 and CBP/p300 bromodomain co-inhibitors.

Targets	Compound	Structure	Functions	References
BD2 bromodomain selective inhibitor	ABBV-744		Induces anticancer effects against acute myeloid leukemia and prostate cancer in vitro and in mouse models with better toxicity profile than BD1 and BD2 bromodomain inhibitors	[55]
BD2 bromodomain selective inhibitor	GSK620		Suppresses inflammatory disease in preclinical models	[58]
PROTAC BRD4 protein degrader	ARV-771		Reduces castration-resistant prostate cancer cell proliferation and survival in vitro, and results in tumor regression in mice.	[60]
PROTAC BRD4 protein degrader	A1874		Combines JQ1 and the MDM2 antagonist idasanutlin activities, degrades BRD4 protein by 98% and stabilizes p53 protein. Reduces cancer cell proliferation and survival.	[61]
BRD4 and CBP/p300 bromodomain co-inhibitor	XP-524		Shows anticancer efficacy comparable to combination therapy with the BRD4 inhibitor JQ-1 and the CBP/p300 inhibitor SGC-CBP30 in pancreatic ductal adenocarcinoma cells.	[62]
BRD4 and CBP/p300 bromodomain co-inhibitor	NEO2734		Show more potent anticancer effects than single-agent BRD4 or CBP/p300 inhibitors alone. Induces colorectal cancer, leukemia and lymphoma cell apoptosis in vitro and in mouse models.	[63, 64]

### Small molecule compound proteolysis-targeting chimera (PROTAC) BRD4 protein degraders in cancer therapy

PROTAC protein degraders are emerging as novel anticancer agents. ARV-771, a small molecule compound PROTAC BRD4 protein degrader, down-regulates the expression of oncogenes such as *MYC* [59]. ARV-771 reduces cell proliferation and induces apoptosis substantially more effectively than the BRD4 inhibitor JQ1 and OTX015 in castration-resistant prostate cancer and diffuse large B cell lymphoma cells [59, 60]. Importantly, while OTX015 suppresses castration-resistant prostate cancer progression, treatment with ARV-771 results in tumor regression in mice xenografted with castration-resistant prostate cancer cell tumors [60] and growth inhibition in mice xenografted with diffuse large B cell lymphoma cells [59] (Table 2).

A1874 is a nutlin-based small molecule compound PROTAC BRD4 protein degrader. A1874 combines the activities of the BRD4 inhibitor JQ1 and the MDM2 antagonist idasanutlin, degrades BRD4 protein by 98% at nanomolar concentrations and stabilizes p53 protein [61]. Treatment with A1874 more significantly reduces cell proliferation and induces cell death in a variety of cancer cell lines with wild type p53 than PROTAC BRD4 protein degraders [61] (Table 2). PROTAC BRD4 protein degraders are therefore likely to be more effective anticancer agents than BRD4 bromodomain inhibitors.

### Small molecule compound dual BRD4 and CBP/p300 bromodomain co-inhibitors in cancer therapy

Another effective approach is to target the bromodomains of the super-enhancer “reader” BRD4 and the “writers” CBP/p300 simultaneously. The dual BRD4 and CBP/p300 bromodomain co-inhibitor XP-524 exhibits higher potency and superior tumoricidal activity than the BRD4 inhibitor JQ-1, and shows anticancer efficacy comparable to combination therapy with high-dose JQ-1 and the CBP/p300 inhibitor SGC-CBP30 in pancreatic ductal adenocarcinoma cells [62]. XP-524 suppresses KRAS activity, blocks KRAS-induced malignant transformation in vivo and improves mouse survival in transgenic mouse models of aggressive pancreatic ductal adenocarcinoma. In addition, XP-524 and an anti-PD-1 antibody exert synergistic anticancer effects and improve survival in two transgenic mouse models of pancreatic ductal adenocarcinoma cells [62] (Table 2).

The other dual BRD4 and CBP/p300 bromodomain co-inhibitor NEO2734 up-regulates the expression of p53 and its target PUMA and induces colorectal cancer cell apoptosis through the intrinsic and extrinsic apoptosis pathways, suppression of the intrinsic or extrinsic apoptosis pathway partly rescues colorectal cancer cells, and NEO2734 represses colon cancer progression by inducing colorectal cancer cell apoptosis in a mouse model [63] (Table 2). In addition, NEO2734 shows more potent anticancer effects than single-agent BRD4 or CBP/p300 inhibitors in lymphoma and acute myeloid leukemia cell lines, and exerts substantial anticancer effects in mouse models of lymphoma and acute myeloid leukemia [64] (Table 2). Dual BRD4 and CBP/p300 bromodomain co-inhibitors are therefore likely to be more effective anticancer agents than BRD4 bromodomain inhibitors.

### BRD4 inhibitors show anticancer effects in clinical trials

More than a dozen BRD4 BD1 and BD2 bromodomain inhibitors, including ABBV-075, AZD5153, BAY 1238097, BMS-986158, BMS-986378, CC-90010, CPI-0610, FT-1101, GSK525762 (Molibresib), INCB054329, INCB057643, ODM-207, OTX015 and PLX51107 have been or are currently in clinical trials in patients with cancer from various organ origins. The BRD4 inhibitors show anticancer effects in clinical trials as monotherapy, but it is now clear that BRD4 inhibitors need to be combined with other anticancer agents to effectively treat cancer patients (Table 3).

**Table 3:** BRD4 BD1 and BD2 bromodomain inhibitors in clinical trials.

BRD4 inhibitor	Cancer type	Trial Phase	Anticancer effects	Side effects	References
OTX015	Lymphoma, myeloma & acute myeloid leukemia	Phase I	A minority of patients achieve complete or partial remission	Thrombocytopenia, diarrhea, vomiting, fatigue, and hyponatraemia. Manageable, progressed into Phase II trials.	[65, 66]
CC-90010 alone, or CC-90010 + Temozolomide	Glioblastomaother solid tumors & lymphoma	Phase I & Ib	Anticancer effects in a minority of patients by CC-90010. Promising anticancer effects by combination therapy	Thrombocytopenia, anemia, and fatigue. Well-tolerated.	[67, 68]
ABBV-075, ABBV-075 plus Venetoclax	Prostate cancer, other solid tumors, acute myeloid leukemia	Phase I	Monotherapy shows limited anticancer effects, ABBV-075 plus Venetoclax is much more effective.	Dysgeusia, loss of appetite, diarrhea, fatigue, nausea, thrombocytopenia, and anemia. Manageable.	[69, 70]
INCB054329 or INCB057643	Solid tumor & lymphoma	Phase I/II	A minority of patients achieve complete or partial remission.	Thrombocytopenia, nausea, fatigue and decreased appetite. Manageable.	[71]
*Pelabresib* (CPI-0610) Plus Ruxolitinib	Myelofibrosis	Phase II & III	The majority of patients achieve partial response.	Thrombocytopenia and anemia. Well-tolerated.	[72, 73]
GSK525762 (*Molibresib*)	NUT carcinoma, leukemia, lymphoma, myeloma	Phase I	A minority of patients achieve complete or partial remission.	Thrombocytopenia, anemia and neutropenia limit dose-escalation and anticancer effects	[74–76]
BAY 1238097	Solid tumors	Phase I	On-target effects on BRD4-inhibition biomarkers, such as reduction in *MYC* expression.	Dose-limiting toxicities including nausea, vomiting, headache, back pain and fatigue. Trial was terminated.	[77]
ODM-207	Solid tumors including prostate cancer	Phase I	No complete or partial responses were observed	Thrombocytopenia, anorexia, nausea, diarrhea and fatigue, indicating a narrow therapeutic window	[78]

In a dose-escalation, phase I clinical study in acute myeloid leukemia, lymphoma and myeloma patients, plasma OTX015 concentration increases proportionally up to 120 mg/day [65, 66]. A minority of patients achieve complete remission or partial remission [65, 66]. While minor side effects, including thrombocytopenia, diarrhea, vomiting, fatigue and hyponatraemia occur, OTX015 is well-tolerated and is currently undergoing phase II clinical trials in patients with acute leukemia, lymphoma or myeloma on a 14 days on and 7 days off schedule (Table 3).

In a Phase I clinical trial of the BRD4 inhibitor CC-90010 in 67 solid tumor and 2 lymphoma patients, one patient each with astrocytoma or endometrial carcinoma achieves a complete response or a partial response, and six additional patients experience prolonged stable disease [67]. Side effects including thrombocytopenia anemia and fatigue are well-tolerated, and CC-90010 at 45 mg on a 4 days on and 24 days off schedule has been proposed for Phase II clinical trials [67] (Table 3). In addition, in a Phase Ib clinical trial in glioblastoma patients, CC-90010 in combination with Temozolomide is safe and well tolerated with encouraging anticancer efficacy [68] (Table 3).

ABBV-075 has been tested in 12 patients with prostate cancer, 72 patients with other solid tumors such as melanoma, colorectal, breast and pancreatic cancers, and 44 patients with acute myeloid leukemia [69, 70]. While ABBV-075 monotherapy shows limited anticancer effects in both solid tumor and leukemia patients, combination therapy with ABBV-075 and the BLC2 inhibitor Venetoclax is considerably more effective. Despite adverse events including dysgeusia, loss of appetite, diarrhea, thrombocytopenia, fatigue, nausea and anemia, ABBV-075 has a good safety profile for Phase II studies at the dose of 1.5 mg daily [69, 70] (Table 3).

In two independent Phase I/II dose-escalation, safety and tolerability studies of the BRD4 inhibitors INCB054329 and INCB057643 in patients with solid tumors or lymphoma, 69 and 134 patients have been recruited to INCB054329 (completed) and INCB057643 (ongoing) studies respectively [71]. Two complete responses and four partial responses have been observed in INCB057643 treatment group; INCB057643 shows a more favorable pharmacokinetic profile than INCB054329; and side effects, including thrombocytopenia, nausea, fatigue and decreased appetite, can be safely managed in both INCB054329 and INCB057643 treated patients [71] (Table 3).

The BRD4 inhibitor Pelabresib (CPI-0610) has shown synergistic anticancer effects, when combined with Ruxolitinib, the current standard of care treatment in myelofibrosis patients, in 84 myelofibrosis patients in a Phase II clinical trial [72]. At 24 weeks, 68% patients reached a reduction in spleen volume of ≥35%, and 56% acquired a reduction in total symptom score of ≥50%. Side effects including thrombocytopenia and anemia are not common and are manageable. Importantly, a double-blinded placebo-controlled Phase III clinical trial is currently ongoing to examine the synergistic anticancer effects of Ruxolitinib and CPI-0610 combination therapy in myelofibrosis patients [73] (Table 3).

The BRD4 inhibitor GSK525762 (Molibresib) has shown promising anticancer effects in a Phase I clinical trial in patients with NUT carcinoma [74, 75]. However, in a dose-escalation Phase I clinical trial of GSK525762 in 87 patients with acute myeloid leukemia, non-Hodgkin lymphoma or multiple myeloma and in a Phase II clinical trial in 24 patients with relapsed/refractory myelodysplastic syndrome or cutaneous T-cell lymphoma, only 6 patients achieved complete response and 7 patients partial responses [76]. Adverse effects such as thrombocytopenia, anemia and neutropenia limit dose escalation and anticancer effects [76] (Table 3).

Two other BRD4 BD1 and BD2 bromodomain inhibitors also show significant toxicity to normal tissues. In the first phase I, open-label, non-randomized clinical trial of the BRD4 inhibitor BAY 1238097 in 8 patients with solid tumors, BAY 1238097 shows on-target effects on BRD4-inhibition biomarkers, such as reduction in *MYC* expression, but results in dose-limiting toxicities including nausea, vomiting, headache, back pain and fatigue, and the study has been terminated [77] (Table 3). In an open-label Phase I clinical trial of the BRD4 inhibitor ODM-207 in 35 patients with solid tumors including castrate-resistant prostate cancer, no complete or partial responses were observed, and side effects such as thrombocytopenia, anorexia, nausea, diarrhea and fatigue were common, indicating that ODM-207 is not efficacious and has a narrow therapeutic window [78] (Table 3).

Importantly, the BRD4 BD2 domain inhibitor ABBV-744, which shows much less toxicity to normal tissues in preclinical models, has also entered a Phase I clinical trial in relapsed or refractory acute myeloid leukemia patients. However, clinical data have not been published.

## Conclusions and future perspective

Characterized by massive histone H3K27 acetylation signal at the loci of cell identity genes and critical oncogenes, super-enhancers are recognized by the BET bromodomain protein BRD4; and super-enhancers and BRD4 play critical roles in oncogene transcriptional activation, over-expression, malignant transformation, cancer cell proliferation, survival, tumor initiation, progression and metastasis in a number cancer types. However, it is important to note that super-enhancers and BRD4 can also activate tumor suppressor gene transcription and suppress drug resistance gene expression. While super-enhancers and BRD4 generally promote tumorigenesis, it is imperative to comprehensively investigate the specific scenarios, such as certain sub-types of cancer cells under particular cellular context, in which super-enhancers and BRD4 exert tumor suppressive, rather than tumorigenic, functions.

BRD4 bromodomain BD1 and BD2 inhibitors have been discovered through small molecule compound library screen, in silico compound screen and chemical synthesis. By blocking BRD4 binding to super-enhancers, BRD4 inhibitors suppress oncogene transcription and expression, reduce cancer cell proliferation and survival, and suppress tumor progress in cancers of a variety of organ origins. However, BRD4 inhibitors, like other targeted therapies, show moderate anticancer effects when employed as a monotherapy. Pre-clinical studies have shown that BRD4 inhibitors exert synergistic anticancer effects when combined with other anticancer agents, such as CDK7 inhibitors, CDK4/CDK6 inhibitors, HDAC inhibitors and BCL-2 inhibitors in vitro and in mouse models of various cancers.

More than a dozen BRD4 BD1 and BD2 bromodomain inhibitors, such as OTX015, have been or are currently in clinical trials in patients with cancer of various organ origins. It is now clear that BRD4 BD1 and BD2 bromodomain inhibitors induce weak to moderate anti-cancer effects in patients as a monotherapy and some of the inhibitors cause significant side effects, such as thrombocytopenia, dysgeusia, diarrhea, fatigue, nausea and anemia. More recently, BRD4 BD2 bromodomain selective inhibitor ABBV-744, PROTAC BRD4 protein degraders such as ARV-771 and A1874, and dual BRD4 and CBP/p300 bromodomain co-inhibitors NEO2734 and XP-524 have been developed and have shown better anticancer effects and/or better safety profile in pre-clinical models. In addition, data from clinical trials of ABBV-744 and NEO2734 are expected to be released, and will further shed lights on the utility of the novel BRD4 inhibitors in the clinical setting.

Future endeavors can focus on developing more potent and selective small molecule compound BRD4 BD2 bromodomain inhibitors to reduce cytotoxicity to normal cells, PROTAC BRD4 protein degraders, and dual BRD4 and CBP/p300 bromodomain co-inhibitors through chemical synthesis, structure-based in silico screen, and wet lab screen of small molecule compound libraries. Their safety profile in normal cells and tissues, pharmacokinetics and anticancer effects can be examined both in vitro and in multiple mouse models. Nevertheless, it should be noted that treatment with BRD4 inhibitors can reduce tumor suppressor gene expression under specific conditions, and that long-term treatment with BRD4 inhibitors can result in cancer cell resistance to a broad spectrum of anticancer agents. It is therefore important to investigate the specific scenarios, such as certain sub-types of cancer cells under particular context and chemotherapy-naïve or -exposed cancer cells and mouse models, in which BRD4 inhibitors reduce tumor suppressor gene expression, augment drug resistance gene expression and render cancer cell resistance to anticancer agents.

As all targeted therapies are expected to be employed in the clinic in combination therapies, the other anticancer agents which exert the best synergistic anticancer effects with BRD4 inhibitors should be identified by unbiased screening of approved anticancer drug libraries against each cancer type. Ultimately, the best combination therapies with BRD4 inhibitors and other anticancer drugs are expected to be tested in clinical trials in patients.
